# The influence of toxic working environment on the urothelial bladder tumors characteristics, the experience of “Sf. Ioan” Clinical Emergency Hospital on selected series

**Published:** 2012-09-25

**Authors:** M Drăguţescu, B Geavlete, R Multescu, B Mihai, C Moldoveanu, P Geavlete

**Affiliations:** “Sf. Ioan” Clinical Emergency Hospital, Department of Urology, Bucharest

**Keywords:** toxic environment, bladder tumor, urothelial carcinoma, transurethral resection

## Abstract

**Introduction: **A correlation between urothelial bladder tumor incidence and exposure to various occupational toxic factors was established for more than a century. The aim of our study was to establish the relationship between these two features.

**Materials and methods: **We analyzed one hundred consecutive bladder tumor patients treated in “Sf. Ioan” Clinical Emergency Hospital, Department of Urology. These cases were studied concerning their occupation and pathological findings.

**Results:**We identified 58 patients having potential urothelial bladder tumors inducing occupations: dye industry, motor vehicle drivers and miners. Among these patients, the incidence of muscle invasive tumors was of 33%, of the high-grade tumors was of 69%, of the multiple tumors was of 60% and of the associated CIS lesions was of 38%. All these rates were significantly lower in the non-occupational hazard group: 12%, 26%, 29% and 14% respectively.

**Conclusions:** Exposure to occupational toxic factors seems to influence the evolution of urothelial bladder tumor into more aggressive patterns. Further studies in this regard are necessary.

## Introduction

Urothelial carcinoma of the transitional epithelium is the most frequent bladder cancer in Europe and in the USA, representing 90-95% of such cases. It occurs mainly in the 6th to 8th decades of life [**1,2**]. The main risk factors are represented by cigarette smoking and occupational exposures frequently encountered in rubber, plastics, dye, textiles, motor vehicle and mining industries. Products such as aniline, benzidine and naphtylamine are usually incriminated, with 20 to 30 years latency after exposure [**3**].

During the last 25 years, a growing body of evidence emerged, indicating that socioeconomic factors also substantially contribute to the etiology of bladder tumors and may play an even more important role than the occupational environment. A number of high- and low-risk industries, occupational exposures and particular occupations were identified to be associated with an elevated risk of bladder cancer. 

The correlation between urothelial bladder tumors’ incidence and exposure to various occupational toxic factors has been established for more than a century. The aim of our study was to evaluate the relationship between the exposure to toxic working environment and urothelial bladder characteristics in our series of patients.

## Patients and methods

We analyzed 100 consecutive bladder cancer patients treated in “Sf. Ioan” Clinical Emergency Hospital, Department of Urology, between January 2010 and August 2010. The aim of the trial was to retrospectively analyze these patients with regard to their occupation and pathological findings. 

The subjects were specifically questioned during admission about the eventual employment in certain industries considered as relevant for the risk of bladder cancer. Also, all available data were gathered concerning the lifelong occupational history, giving special attention to specific exposures to fumes, dusts, smoke and chemicals. In addition, the patients provided details on past medical and residential history.

The investigation protocol consisted of urinary cytology, abdominal ultrasound, intravenous pyelography or computer tomography.

**Fig. 1:**
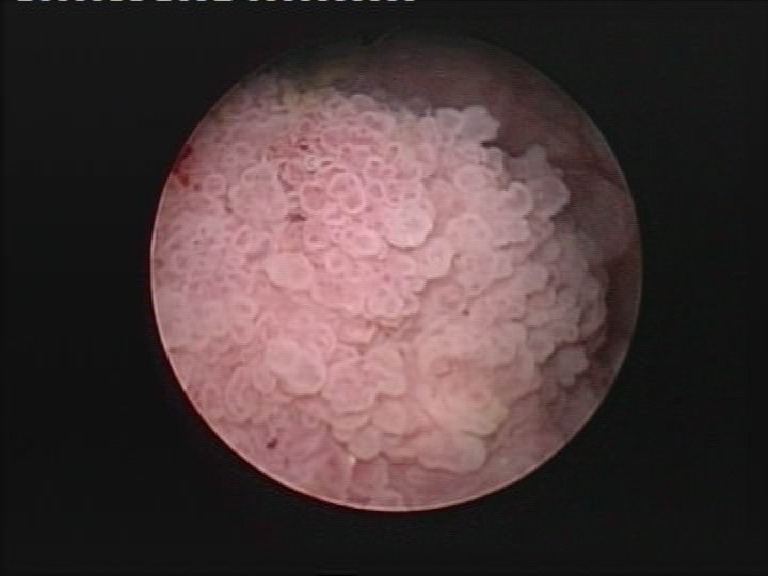
Urothelial bladder tumor

Monopolar transurethral resection of bladder tumors (TURBT) or transurethral resection in saline (TURis) was used as first line endoscopic treatment. Also, hexaminolevulinate blue light cystoscopy (HAL-BLC) or narrow band imaging (NBI) cystoscopy was also applied while aiming to improve the bladder tumors diagnostic accuracy. 

**Fig. 2:**
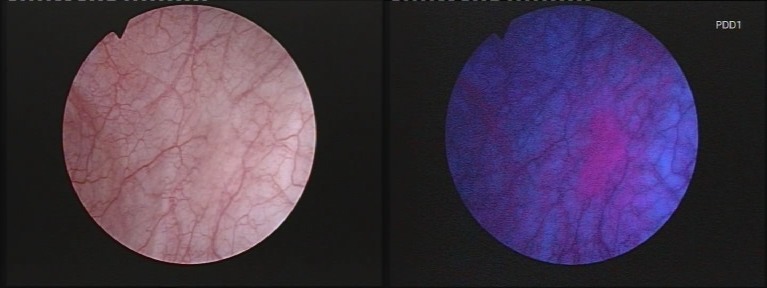
CIS lesion in white light (left) and blue light (right)

**Fig. 3:**
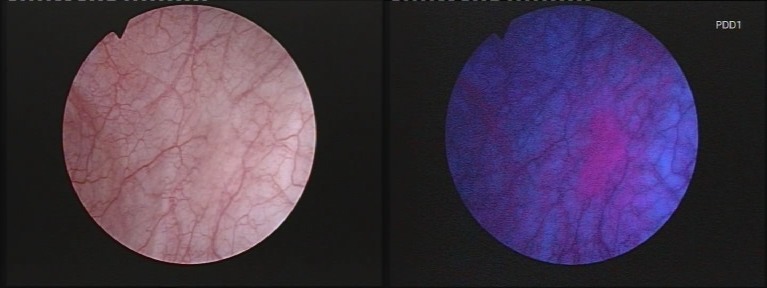
pT1 tumor in white light (left) and NBI (right)

The histology form, containing confirmation of case histology and date of diagnosis, was completed from hospital records. The statistical analysis was performed with Chi-squared test, with the cut-off value for statistical significance p<0.05.

## Results

The mean age at diagnosis was of 67 years (range 45-89) and the male/female ratio was of 3:1. Most patients had a smoking history and a total of 21% of the cases associated more than 20 years of occupational exposure (**Table 1**).

**Table 1:** Patient characteristics

Feature	N (%)
Mean age	67 (45-89)
Gender	
Male	71 (71%)
Female	29 (29%)
History of smoking	74 (74%)
>20 years of occupational exposure	21 (21%)

We identified 58 patients potentially presenting urothelial bladder cancer inducing occupations: dye industry, motor vehicle drivers and miners. Among these cases, the incidence of muscle invasive tumors was of 33%, of high-grade tumors was of 69%, of multiple tumors was of 60% and of the associated CIS lesions was of 38%. All these rates were significantly lower in the non-occupational hazard group: 12%, 26%, 29% and 14%, respectively (**Table 2**).

**Table 2:** The incidence of bladder tumors depending on occupational exposure

	Occupational hazard group	Non-occupational hazard group	p - value
Muscle invasive tumors	33% (19/58)	12% (5/42)	p – 0.00037
High-grade tumors	69% (40/58)	26% (11/42)	p < 0,0001
Multiple tumors	60% (35/58)	29% (12/42)	p < 0.0001
CIS lesions	38% (22/58)	14% (6/42)	p < 0.0001

## Discussion

In most studies concerning the influence of occupational exposure on bladder cancer incidence, at least 20-25% of bladder tumors are related to occupational exposure.

In the first half of this century, such exposures to certain aromatic amines and amides were the first to be established as carcinogenic for the urinary bladder epithelium. In addition, the past 30 years have seen quite a number of reports constantly showing an increased bladder cancer risk linked to the use of cigarettes. Since the original associations between occupational exposures and bladder cancer occurrence were observed for synthetic dye workers, it could be expected that observational studies over the past 20 years would still emphasize an increased risk for these individuals. This perspective seems to be confirmed by most of the available studies [**4,5**].

The occupational use of dyes is also common within both the textile and leather industries. However, the relation between the bladder cancer risk and such exposures is less clear than for those described within the dye industry. Other recent case-control studies reported significantly increased risks (between 3.1 and 8.8 times) among workers in the dyestuffs industry [**4,6,7**].

Another occupational exposure possibly related to a substantial risk is that to polycyclic aromatic hydrocarbons (PAHs), which may appear as products of coal or petroleum combustion. PAHs are prevalent in motor vehicle exhausts, and several studies described a significantly increased risk for individuals occupationally exposed to diesel or traffic fumes (such as truck, bus or taxi drivers and motor mechanics) [**8,9**].

Increased risks for truck, bus, and locomotive drivers were reported in most studies [**9,12**]. The higher bladder cancer risk for truck drivers may come from the inhalation of motor exhaust emission particles in the truck as well as from on-the-road exposure to such substances [**10,13**]. This view is supported by the observation that jobs associated with an exposure to diesel or traffic fumes are linked with an elevated risk of bladder cancer [**6**].

Another possible explanation is constituted by the less frequent micturition typical for these occupation categories, subsequently leading to urinary retention and a higher urine pressure. These specific features result in a more intensive and prolonged contact of potential urine-born carcinogenic agents with the urothelium. Another setting in which PAH exposure might occur is represented by the occupational contact with coal, tars and asphalt in particular jobs such as coal mining, coke oven operations, roofing and paving. Some trials emphasized the significantly higher risks related to such exposures [**14**].

According to a Canadian study, miners, drillers and blasters presented an odds ratio of 4.5 as well as a relative risk estimation increased with the employment duration (over 10 years, OR 8.1) [**15**]. Another trial reported a twofold OR for working in the mining industry, which did not show a risk trend proportional with the employment length [**16**]. Increased risks of bladder cancer among miners were repeatedly reported in the literature [**6-10,16-20**]. Diesel equipment is commonly used in the above ground and underground operations, as are drilling devices with cutting oils.

Although the correlation between the incidence of urothelial bladder tumors and the exposure to various factors was clearly established, none of the studies determined a connection between tumor histology and occupational toxic factors. However, it seems that the exposure to the above-mentioned elements leads to a more aggressive histological pattern. We found the incidence of muscle invasive tumors, high-grade tumors, multiple tumors and associated CIS lesions to be significantly higher in the occupational toxic factors’ group.

## Conclusions

The exposure to occupational toxic factors seems to influence the evolution of urothelial bladder tumors into more aggressive patterns. Further studies are required in order to clarify this perspective. Such trials could increase our understanding of the multifactorial, multisequential, and multistage tumor formation process in the lower urinary tract, thus leading to preventive measures aiming to reduce the incidence of bladder cancer.

## References

[1] Jemal  A, Bray  F, Center  MM (2011). Global cancer statistics. CA Cancer J Clin.

[2] Siegel  R, Ward  E, Brawley  O (2011). The impact of eliminating socioeconomic and racial disparities on premature cancer deaths. CA Cancer J Clin.

[3] Boffetta  P, Jourenkova  N, Gustavsson  P (1997). Cancer risk from occupational and environmental exposure to polycyclic aromatic hydrocarbons. Cancer Causes Control.

[4] Vineis  P, Magnani  C (1985). Occupation and bladder cancer in males: a case-control study. Int J Cancer.

[5] Cole  P, Hoover  R, Friedell  GH (1972). Occupation and cancer of the lower urinary tract. Cancer.

[6] Risch  HA, Burch  JD, Miller  AB (1988). Occupational factors and the incidence of cancer of the bladder in Canada. Br lndustr Med.

[7] Bonassi  S, Merlo  F, Pearce  N (1989). Bladder cancer and occupational exposure to polycyclic aromatic hydrocarbons. Int f Cancer.

[8] Coggon  D, Pannett  B, Osmond  C (1986). A survey of cancer and occupation in young and middle aged men. II. Non respiratory cancers. Br J Ind Med.

[9] Silverman  DT, Hoover  RN, Mason  TJ (1986). Motor exhaust-related occupations and bladder cancer. Cancer Res.

[10] Schifflers  E, Jamart  J, Renard  V (1987). Tobacco and occupation as risk factors in bladder cancer: A case-control study in southern Belgium. Int J Cancer.

[11] Jensen  OM, Wahrendorf  J, Knudsen  JB (1987). The Copenhagen case-referent study on bladder cancer. Risks among drivers, painters and certain other occupations. Scand J Work Environ Hea.

[12] Wynder  EL, Dieck  GS, Hall  NEL (1985). A case-control study of diesel exhaust exposure and bladder cancer. Environmental Res.

[13] Theriault  G, Tremblay  C, Cordier  S (1984). Bladder cancer in the aluminium industry. Lancet.

[14] Teschke  K, Morgan  MS, Checkoway  H (1997). Surveillance of nasal and bladder cancer to locate sources of exposure to occupational carcinogens. Occupational and Environmental Medicine.

[15] Kunze  E, Chang-Claude  J, Frentzel-Beyme  R (1992). Life style and occupational risk factors for bladder cancer in Germany. A case-control study. Cancer.

[16] Anthony  HM, Thomas  GM (1970). Tumors of the urinary bladder: an analysis of the occupations of 1030 patients in Leeds, England. J Nad Cancer Inst.

[17] Brownson  R, Chang  J, Davis  J (1987). Occupation, smoking, and alcohol in the epidemiology of bladder cancer. Am Jf Public Health.

[18] Dolin  P (1992). A descriptive study of occupation and bladder cancer in England and Wales. BrJ Cancer.

[19] Howe  GR, Burch  JD, Miller  AB (1980). GM, Morrison B, Gordon P, et al. Tobacco use, occupation, coffee, various nutrients, and bladder cancer. Jf Natd Cancer Inst.

[20] Cordier  S, Clavel  J, Limasset  JC (1993). Occupational risks of bladder cancer in France: a multicentre case-control study. Int J7 Epidemiol.

